# Supplementary material for: Fitness Costs of the Glutathione S-Transferase Epsilon 2 (L119F-GSTe2) Mediated Metabolic Resistance to Insecticides in the Major African Malaria Vector Anopheles Funestus
Source: Genes (Basel). 2018 Dec 19;9(12):645. doi: 10.3390/genes9120645 (PMC6316527; doi:10.3390/genes9120645)
Supplement: Supplementary file 1 [file genes-09-00645-s001.zip › Supplementary table S2.pdf]

Table S2: Change in the distribution of L119F-GSTe2 genotypes and pupae formation

Genotypes	*Pupae D9 vs. Pupae D11*		*Pupae D11 Vs. Pupae D13*	
	**Chi-square**	***P*-value**	**Chi-square**	***P*-value**
**RR**	3.24	0,07	2.33	0.12
**RS**	8.96	0.0028*	2.34	0.12
**SS**	3.65	0,03*	0.32	0.50
