# Supplementary material for: Fitness Costs of the Glutathione S-Transferase Epsilon 2 (L119F-GSTe2) Mediated Metabolic Resistance to Insecticides in the Major African Malaria Vector Anopheles Funestus
Source: Genes (Basel). 2018 Dec 19;9(12):645. doi: 10.3390/genes9120645 (PMC6316527; doi:10.3390/genes9120645)
Supplement: Supplementary file 1 [file genes-09-00645-s001.zip › Supplementary table S1.pdf]

Table S1: Distribution of L119F-GSTe2 genotypes between oviposited and non oviposited females.

	**Oviposited females**	**Oviposited females**
Genotype		
L119F-RR	16 (6.15)	10 (10)
L119F-RS	97(37.31)	43(43)
L119F-SS	147 (56,54)	47 (47)
Allele		
119F-R	24.80%	31.50%
L119-S	75.20%	68.50%
